# HighVia—A Flexible Live-Cell High-Content Screening Pipeline to Assess Cellular Toxicity

**DOI:** 10.1177/2472555220923979

**Published:** 2020-05-27

**Authors:** Alison Howarth, Martin Schröder, Raquel C. Montenegro, David H. Drewry, Heba Sailem, Val Millar, Susanne Müller, Daniel V. Ebner

**Affiliations:** 1 Nuffield Department of Clinical Medicine, Target Discovery Institute, University of Oxford, Oxford, UK; 2 Structural Genomics Consortium, Buchmann Institute for Molecular Life Science, Institute for Pharmaceutical Chemistry, Johann Wolfgang Goethe-University, Frankfurt, Germany; 3 Nuffield Department of Clinical Medicine, Structural Genomics Consortium, University of Oxford, Oxford, UK; 4 Federal University of Ceará, Drug Research and Development Center (NPDM), Pharmacogenetics Laboratory, Fortaleza, CE, Brazil; 5 Structural Genomics Consortium, UNC Eshelman School of Pharmacy, University of North Carolina at Chapel Hill, Chapel Hill, NC, USA; 6 Department of Engineering, University of Oxford, Oxford, UK; * These authors contributed equally.

**Keywords:** high content, high throughput, viability, apoptosis, multiplex

## Abstract

High-content screening to monitor disease-modifying phenotypes upon small-molecule addition has become an essential component of many drug and target discovery platforms. One of the most common phenotypic approaches, especially in the field of oncology research, is the assessment of cell viability. However, frequently used viability readouts employing metabolic proxy assays based on homogeneous colorimetric/fluorescent reagents are one-dimensional, provide limited information, and can in many cases yield conflicting or difficult-to-interpret results, leading to misinterpretation of data and wasted resources.The resurgence of high-content, phenotypic screening has significantly improved the quality and breadth of cell viability data, which can be obtained at the very earliest stages of drug and target discovery. Here, we describe a relatively inexpensive, high-throughput, high-content, multiparametric, fluorescent imaging protocol using a live-cell method of three fluorescent probes (Hoechst, Yo-Pro-3, and annexin V), that is amenable to the addition of further fluorophores. The protocol enables the accurate description and profiling of multiple cell death mechanisms, including apoptosis and necrosis, as well as accurate determination of compound IC_50_, and has been validated on a range of high-content imagers and image analysis software. To validate the protocol, we have used a small library of approximately 200 narrow-spectrum kinase inhibitors and clinically approved drugs. This fully developed, easy-to-use pipeline has subsequently been implemented in several academic screening facilities, yielding fast, flexible, and rich cell viability data for a range of early-stage high-throughput drug and target discovery programs.

## Introduction

High-throughput screening (HTS) is an essential component of fully integrated drug and target discovery screening regimes, with high-content screening (HCS) becoming an increasingly important part of the discovery process, from early-stage development and screening to hit validation and candidate selection.^1^ Cell-based HCS provides data-rich, physiologically relevant information that has proven to be a powerful tool in the search for high-quality small-molecule leads.^2^ The power of HCS originates from the fact that cell imaging, using multiplexed fluorescent probes targeting distinct cellular structures or events, allows characterization of complex phenotypes that are directly relevant to disease pathology, including cell stress pathways and cell death induction. HCS can also facilitate early absorption, distribution, metabolism, and excretion–toxicity (ADMET) profiling, as well as target the prediction of a novel candidate molecules.^3^

In the context of oncology screening, correctly assessing cell viability and determining mechanisms of cell death is critically important. Standard methods of assessing cell viability commonly utilize metabolic proxy assays including tetrazolium-based assays (e.g., MTT) and resazurin or total protein assays, such as streptavidin-based systems (SBSs).^4^ Although easy to set up and inexpensive, such readouts are limited, providing only a simplistic, one-dimensional readout of complex, heterogeneous cell populations that fails to accurately reflect a comprehensive range of potential cellular phenotypes, including the induction of quiescence, senescence, cellular stress, and cell death mechanisms. Because they do not provide detailed information on the mechanism of action, they often produce misleading or invalid data, which can consume considerable time and resources validating spurious primary screening results.

Additionally, these assays are often susceptible to environmental factors including cell density, nutrient availability in growth media, pH, compound–substrate interactions, toxicity, constraints of reagent turnover, refeeding, and multiple read points over long-ranging assays, which can diminish their utility and physiological relevance. In our experience, and the experience of others,^5-7^ these factors require significant validation, especially when investigating metabolic or radiotherapeutic targets for cancer therapy where mutations in mitochondrial DNA, dysfunctional metabolic enzymes, and deregulated gene expression leading to altered^8^ metabolic function can severely compromise metabolic proxy assays that monitor metabolic activity via oxidative phosphorylation (OXPHOS).

Because of the limitations of homogenous, metabolic proxy assays, especially in the field of oncology-based screening, we initiated the development of a high-throughput/high-content assay with the goal of measuring cell viability and proliferation while simultaneously investigating cell death mechanisms. Several HCS methods exist, ranging from the very powerful “Cell Painting” technique^9^ for morphological profiling of small compounds, functional pathway grouping, and identification of signatures of disease, to simpler protocols using two fluorophores to monitor cell viability mechanisms.^10^ While Cell Painting requires significant downstream data evaluation efforts, simpler methods do not have the capacity to measure cell viability and proliferation while simultaneously investigating cell death mechanisms, in a cost-effective manner, as a live-cell protocol.

Here, we describe a fast, flexible, and relatively inexpensive multiplexed HTS protocol employing the simplicity of an add-mix-read three-stain multiparametric method. This optimized high-content imaging (HCI)-based method avoids many of the issues observed in metabolic proxy assays, including potential artifacts generated by compromised metabolic pathways in cancer cell models, by providing direct single-cell analysis. We have validated the protocol against several cancer cell lines of different tissue origin using an FDA-approved oncology drug panel comprising a range of mechanisms of action as well as a small library of kinase inhibitors. The protocol produces a robust and reproducible high-content readout for cell viability across different cancer cell lines while simultaneously profiling heterogeneous cell populations for small-molecule induced toxicity mechanisms, including apoptosis at the early induction phase or late stage, and necrosis. Further flexibility is offered in this protocol as additional fluorophores or expression vectors can be added in the available spectrum (**Suppl. Fig. S1**), including caspase 3/7, mitochondrial potential dyes, or LC-3 autophagy. This depth of analysis can provide increased accuracy in determining cellular pathway induction. Image acquisition and profiling of populations were validated in three independent HCS imaging systems with three image analysis software methods for profiling populations.

## Materials and Methods

### Compound Library Preparation

The compound stocks of an FDA-approved oncology small-compound library, containing 101 compounds representing a range of mechanisms of action (**Suppl. Table S1**), were dissolved in DMSO to a concentration of 10 mM. Using an Echo 550 (LabCyte, San Jose, California, USA), we created intermediate 384-well working stock plates containing 0.48 µL of either 101 individual (single-point) compounds or two replicates of 10 target-specific clinically approved compounds with a 7-point 1:2 serial dilution (**Suppl. Table S1**). Intermediate working stock plates were diluted with 80 µL of Opti-MEM (Thermo Fisher) to a concentration of 60 μM for single-point compounds and 60–0.9 μM for serial dilutions. The Kinase Chemogenomic Set (KCGS) library, a well-annotated set of 188 potent and selective small-molecule inhibitors that target 221 kinases and function as a valuable tool for facilitating target deconvolution, was obtained from the Structural Genomics Consortium, University of North Carolina (SGC-UNC) already assembled in a plate format suitable for the Echo acoustic dispenser system at 10 mM DMSO stock concentration (https://www.sgc-unc.org/kcgs). Compounds were dispensed directly into cell plates using the Echo acoustic dispenser to achieve a 10 μM final concentration.

### Cell Screening Liquid Handling Protocol

HeLa and AGP-01 cells were plated at a density of 600 (24 and 48 h time points) or 300 (72 h time point) cells/well, while MDA-MB-231 cells were plated at a density of 2000 (24 and 48 h time points) or 1000 (72 h time point) cells/well. PANC-1 cells were plated at a density of 500 cells/well. For all cell lines, cell seeding densities were optimized to achieve 70% confluence at the endpoint/readout for both 96- and 384-well plates to permit optimum cell segmentation for analysis. Cells were plated in a volume of 55 µL of culture medium/well in 384-well flat-bottom plates (CellCarrier; PerkinElmer) and incubated for 24 h prior to the addition of 5 µL of compound, using a Janus MDT automated liquid handler (PerkinElmer). For PANC-1 cells, the compound was directly transferred to the individual wells using an Echo acoustic dispenser. Cells were incubated with compound at 37 °C at concentrations of 10–0.9 μM for 24, 48, and 72 h prior to staining and experiment evaluation. The compound library contained compounds with varied mechanisms of action. As such, endpoints/readouts at 24, 48, and 72 h were selected for all compounds and cell lines to capture both rapid and slow-onset cell death mechanisms.

To minimize the effect of DMSO toxicity, a final concentration of 0.05% DMSO was used, which showed little to no effect on cell proliferation.^11^ Control wells containing no DMSO and 0.05% DMSO were included in all plates and used for normalization of the data, thus providing an internal control for DMSO sensitivity of the cells. DMSO concentration was the same for all wells, independent of the concentration.

### HighVia Labeling Protocol

To retain all cells for imaging, including loosely adherent and floating cells, plates were centrifuged at 400*g*/3 min, and 20 µL media was removed at an aspiration rate of 3 μL/s without disturbing the monolayer and replaced with 20 μL/well staining media (Hoechst 33342, 1 μM; Yo-Pro-3, 1 μM; and annexin V Alexa 488, 0.3 μL/well diluted in complete media) (excitation [Ex] and emission [Em] in **Suppl. Table S2** and staining optimization in **Suppl. Fig. S2**). Plates were spun briefly and incubated for 1 h at 37 °C before imaging on an HCI system. Cells are viable for at least 4 h poststaining, and all plates were routinely imaged between 1 and 2 h poststaining. All stains were purchased from Life Technologies (UK) and diluted in phenol-free Dulbecco’s modified Eagle’s medium (DMEM; Sigma).

### Resazurin Whole-Well Viability Assay

Following HCI, staining media was removed without disturbing the monolayer and replaced with 50 µL of resazurin (10 ng/mL; Sigma) in phenol-free media. Plates were incubated for 2 h at 37 °C, prior to fluorescent reading on an Envision multiformat plate reader (PerkinElmer) at Ex 579 nm/Em 584 nm for comparison between cell viability protocols.

### Fluorescent Image Acquisition

#### Operetta (PerkinElmer)

Image acquisition was performed using a 10× air objective, collecting two fields per well: digital phase contrast, 25 ms, transmitted light at 50%; brightfield, transmitted light at 50%; Hoechst, 35 ms exposure, Ex 360–400 nm/Em 410–480 nm; annexin V Alexa 488, 45 ms exposure, Ex 460–490 nm/Em 500–550 nm; and Yo-Pro-3, 35 ms exposure, Ex 560–580 nm/Em 650–760 nm.

#### IN Cell Analyzer 6000 (GE)

Image acquisition was performed, using a 20× 0.45NA plan Fluor ELWD air objective, collecting four fields of view and the following settings (excitation laser followed by emission filter and exposure time): Hoechst staining, UV_DAPI 500 ms; annexin V Alexa 488, Blue_FITC 1500 ms; and Yo-Pro-3, Red_Cy5 1000 ms and TL_Brightfield_DsRed 800 ms.

#### CQ1 (Yokogawa)

Image acquisition of one field per well was performed using a 10× dry microscope (UPLSAPO10). The following parameters were used: for brightfield images, transmitted light at 70%, 50 ms exposure; for Hoechst staining, Ex 405 nm at 50%, Em 447/60, exposure 100 ms; for annexin V Alexa 488 staining, Ex 488 nm at 40%, Em 525/50, exposure 50 ms; and for Yo-Pro-3 staining, Ex 561 nm at 40%, Em 617/73, exposure 100 ms.

The expected throughput for 384-well-format whole-well homogenous readouts of cell viability employing high-throughput four-camera high-content imagers can be 45–60 min/plate.

### Fluorescent Image Segmentation

**Figure 1.**
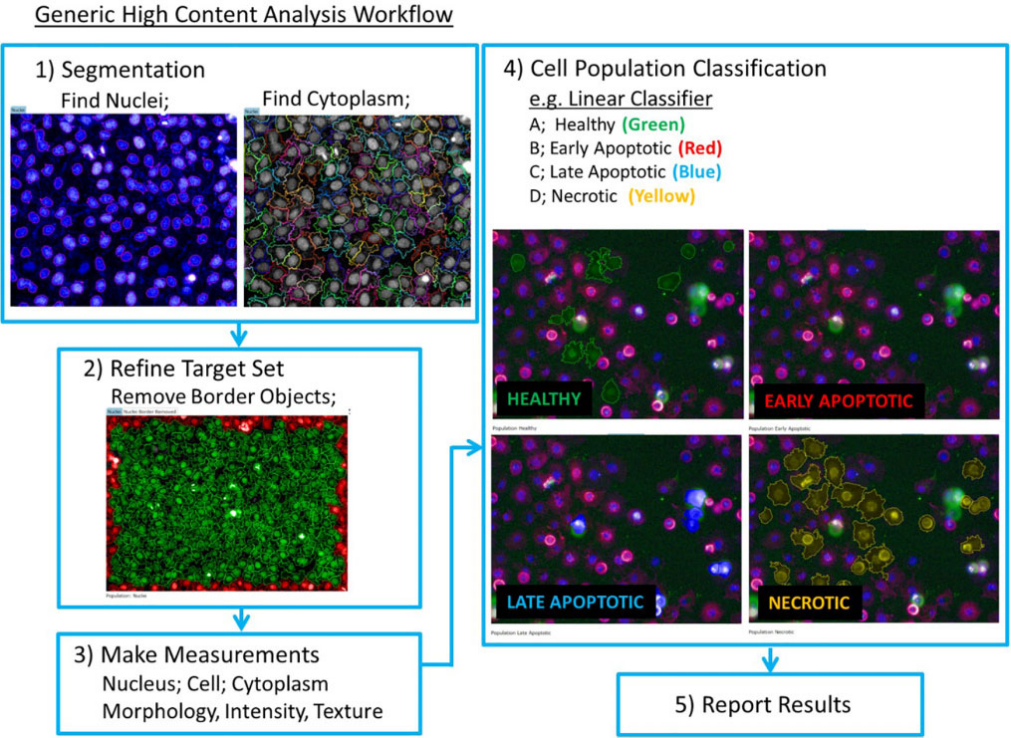
Generic workflow for high-content image segmentation.

Full details of image segmentation are described in **Supplemental Tables S3–S5** with a workflow detailed in **Figure 1**. Briefly, “nuclei” were identified using Hoechst intensity and area to segment regions on the image belonging to cell nuclei. Where required, an increased splitting value was applied to accurately segment “stuck” nuclei, where two or more nuclei lie in close proximity.

Cytoplasmic regions were determined using previously defined “nuclei” (Hoechst) as the starting point; the region “cytoplasm” must be linked to defined nuclei to be detected. It is critical to define the cytoplasm region surrounding defined nuclei with high confidence at this stage.

These image analysis steps create three distinct cell regions for analysis: “nuclei,” “cytoplasm,” and “cell,” respectively. A fourth region of “nuclei border eroded” was created, comprising the “nuclei” region with a 5% erosion from the outer border, providing increased confidence in selecting features within the nuclei region.

Any cells with nuclei on the image border were removed from analysis to ensure that only whole cells were analyzed. For each of the four regions, “nuclei,” “cytoplasm,” “cell,” and “nuclei border eroded,” a comprehensive list of measurements were collected: morphology (e.g., area and roundness), intensity (e.g., mean intensity and standard deviation of intensity within the object), and texture (e.g., SER features—spots, edges, and ridges).

### Classification of Cell Death Modalities

To enable automated and robust identification of different cell death modalities, three techniques were employed; the first was a simple gating/decision tree protocol using intensity and morphology features, the second a machine learning linear classifier called PhenoLOGIC (PerkinElmer), and the third a supervised classification. The linear classifier and supervised classification techniques employ user-defined cell population annotation to allow each software package to combine the most meaningful parameters to achieve accurate classification of cells. Three techniques enable the end user to evaluate each classification technique and adopt or modify the technique most suitable for their application. A detailed discussion of each technique is found below.

### Data Analysis and Statistics

Using HCI data and resazurin, a concentration at which 50% growth inhibition (IC_50_) occurred was calculated for each cell line in GraphPad Prism 5 (La Jolla, CA) and displayed as the percentage of healthy cells within the population. Statistical significance was calculated in GraphPad Prism using an unpaired *t* test. Any *p* values of ≤0.05 were considered statistically significant.

## Results and Discussion

### Development and Optimization of Live-Cell HCI Assay, HighVia

Cell death is a highly complex, dynamic process influenced and induced by multiple pathways with a high degree of molecular cross-talk.^12^ An effective means for determining cellular response to small-molecule treatment is through HCI, which allows image capture at multiple drug concentrations and time points, combining features of multiple viability assessments in a single well, rather than “single-take,” late-stage, static readout viability assays. The early application of an HCI protocol in cell viability screening allows rapid profiling of known and unknown compounds and offers an early-stage, robust validation step for candidate selection whereby ineffective or off-target toxic compounds may be rapidly de-selected. Large primary screens can routinely employ small compound libraries of >500,000 compounds, even in the academic screening setting,^13^ often yielding several hundred positive “hits.” HCI is one way to help sort through and prioritize a hit list from an HTS. HCI can also be used in lead optimization programs to further understand the mechanism of action of key compounds. HCI is being applied in the late stages of discovery programs to ensure that drug candidate molecules do not have inappropriate toxicities or surprising unwanted effects.

It is from this context of broad-scale utility that we sought to implement a robust, easy-to-use, and information-rich HCI protocol. To construct a high-content, multiparametric, fluorescent imaging protocol capable of profiling multiple cellular mechanisms in the same well, it was critical to consider cost, efficiency, throughput, and, above all, optimization of extracting the maximum information available per well.

The first step of our protocol involved mapping the available excitation and emission spectra using Spectra Viewer^14^ to the filter sets available in the PerkinElmer Operetta (**Suppl. Fig. S1**). We were able to define four distinct excitation and emission paired wavelength regions, allowing for sufficient separation between probes (**Suppl. Fig. S1**). The protocol has originally been set up on a PerkinElmer Operetta. However, the established parameters were easily adapted to other HC imagers, such as the Yokogawa CQ1 or the GE IN Cell Analyzer 6000.

Critical to the staining protocol was accurate segmentation of nuclei and cell membrane using live-cell stains. Hoechst was selected to stain nuclei at the outset. To determine necrotic cells, Yo-Pro-3 was used, which is impermeable to cells with intact nuclear membranes. This provided a two-color fluorescent readout to determine dead or live cells but lacked the required sensitivity to discriminate more subtle cell death mechanisms.

To accurately segment the cellular membrane, Life Technologies CellTracker Green CMFDA and CellTracker Violet BMQC CellTrace CFSE (Green) Cell Proliferation Kit and CellTrace Violet Cell Proliferation Kit were explored. Both dyes permeate the cell membrane and are cleaved to a cell-impermeable fluorescent product. CellTrace offers a more brilliant signal than CellTracker, although both produce single-peak fluorescence and are highly suited to multiplexing. We found that the staining was very weak in serum containing media and exhibited high background after staining, requiring media changes before and after staining. For these reasons, CellTracker and CellTrace were excluded from use in the staining set. Instead, by simply increasing the incubation time with Hoechst from 1 to 2 h, we were able to accurately segment both the nuclei and external membrane using intensity thresholding, rendering the need for a specific, and costly, cytoplasm staining dye unnecessary.

Next, we began to investigate dyes specific to apoptosis. Caspase and phosphotidylserine (PS) directed dyes are most commonly used and available as no-wash, live-cell dyes. We first tested CellEvent Caspase-3/7 Green Detection Reagent (Life Technologies). The bright fluorescence allows for easy detection, and the dye requires no wash steps, retaining fragile or detached cells. Although caspase kits are widely used, the sensitivity to determine early and late apoptosis was not achievable, and we therefore tested PS detection using the annexin V Alexa 488 conjugate as an apoptosis marker.

We also investigated the use of tetramethylrhodamine methyl ester perchlorate (TMRM) for assessing mitochondrial activity. TMRM is readily sequestered into active (depolarized) mitochondria and is a useful measure of apoptotic induction and mitochondrial health. Initial experiments gave promising results; however, to retain the lowest costs and most streamlined image analysis protocol, the use of TMRM was subsequently omitted from HighVia, but could easily be added to the protocol if desired.

Finally, we used the brightfield transmitted light to capture cell shape. This was not used for image analysis but provided a useful visual check to ensure accurate image segmentation. The use of digital phase or contrast imaging may allow label-free segmentation techniques to be developed; this is to be investigated in more detail in the future.

### Cell Population Classification Methods.

For reference image details, see **Figure 1**.

#### Classification 1: Population profiling—population gating using a decision tree (GE Investigator 1.6.2; multitarget analysis protocol or Yokogawa Pathfinder software)

The simplest method to profile cell populations was to apply a gating protocol to classify cells into populations according to Yo-Pro-3 and annexin V stain intensity and localization within cells. Nuclear and cytoplasm regions were segmented using the Hoechst channel. Measurements of staining intensity, object/background intensity ratios, localization, texture, and morphology were profiled for Hoechst, Yo-Pro-3, and annexin V channels for each defined region of the cell. Characterization of Yo-Pro-3 staining within the “nucleus” region and annexin V staining within the “whole cell” region (cytoplasm and nuclear region combined) were used to determine the population classifier for each cell.

For the gating procedure, cells were initially sorted into “late and necrotic” or “healthy and early” populations by plotting histograms of nuclear intensity that were Yo-Pro-3 positive against the nuclear area. Cells with a Yo-Pro-3 intensity twofold above that of the background in the “nucleus” region were labeled as the “late and necrotic” population; cells with a Yo-Pro-3 intensity <2-fold above that of the background were labeled as the “healthy and early” population.

The “late and necrotic” and “healthy and early” populations were then gated into two further populations. The “late and necrotic” population was gated according to annexin V presence or absence in the “whole cell” region. Cells with an annexin V intensity >2-fold above the background in the “whole cell” region were labeled “late apoptotic,” and the remaining cells were labeled “necrotic.” The “healthy and early” population was likewise gated according to the presence or absence of annexin V in the “whole cell” region. Histograms of annexin V intensity in the “whole cell” region were plotted against the Hoechst channel nuclei area. Cells with an annexin V staining intensity >2-fold above that of the background in the “whole cell” region were labeled “late apoptotic,” and the remaining cells were labeled “necrotic.” Image labeling should be checked carefully against image sets to ensure accurate labeling; adjustments should be made accordingly and checked between different time points and cell types. This simple method is used to rapidly assess cell death mechanism induction in small-compound screens. A more detailed profiling can be achieved as described below.

#### Classification 2: Population Profiling—Linear Classifier (PerkinElmer)

After the initial segmentation of nuclear and cytoplasm boundaries, features of staining intensity, localization, texture, and morphology were profiled within each region. Characterization of Yo-Pro-3 staining within the “eroded nucleus” region and annexin V staining within the “cytoplasm and nuclei” region was also determined. Using PhenoLOGIC (PerkinElmer), a training set of reference images containing at least 40 cells representative of each of the populations, we manually selected healthy, early, late, and necrotic populations. These reference image sets were used by the Columbus linear classification algorithm to profile the remaining images into the four defined populations.

##### Population Healthy

**Figure 2.**
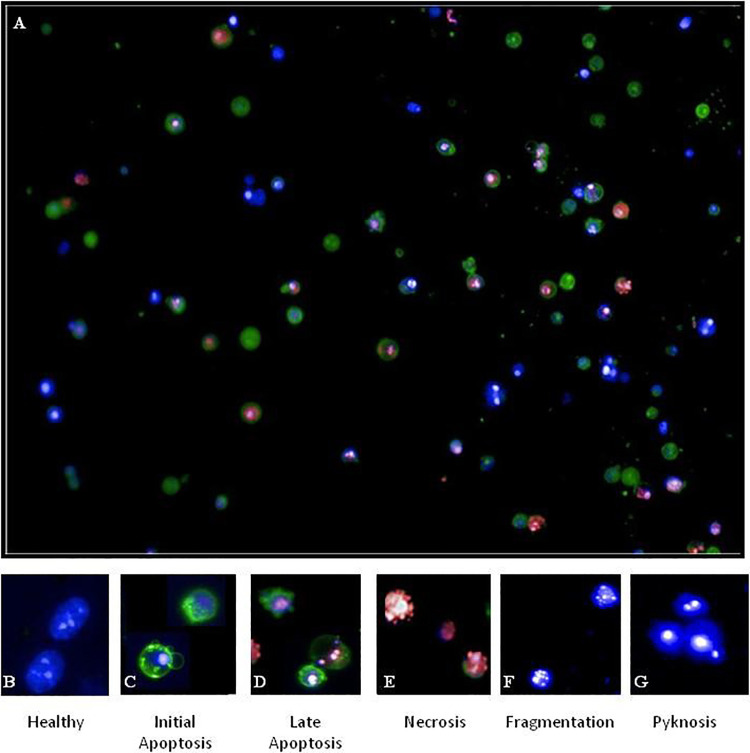
(**A**) Induction of apoptosis following 100 nM staurosporine exposure for 72 h. Distinct features of cellular classification: (**B**) healthy, (**C**) initial apoptosis, (**D**) late apoptosis, and (**E**) necrosis. Nuclei features: (**B**) healthy, (**F**) fragmentation, and (**G**) pyknosis.

Reference images selected from DMSO control wells to determine cellular features of healthy cell size, shape, and Hoechst intensity features were used. Features of healthy cells were a Yo-Pro-3 intensity <2-fold above the background within the “nucleus” region and an annexin V intensity <2-fold above the background within any part of the cell, while maintaining a distinct nuclear/cytoplasm ratio relationship. The circularity of the cells and nuclear region should be <0.8 of the nuclear area, and the intensity should be within normal range for the cell lines. This can be determined by plotting histograms of individual features and excluding any cells that fall within extreme regions of the plots. Labeling should be checked against reference images (**Fig. 2**) for accuracy.

##### Population Initial Apoptos

Features of initial apoptosis were Yo-Pro-3 staining <2-fold above the background within the “eroded nucleus” region; annexin V intensity >2-fold above the background within the “whole cell” region; nuclear collapse to small, homogenous, and intensely stained region; collapse of the nuclear/cytoplasm ratio; and irregular cytoplasm shape (circularity score of <0.8) with membrane blebbing.

##### Population Late Apoptosi

Features of late apoptosis were intense Yo-Pro-3 staining within the “eroded nucleus” region; a minimum Yo-Pro staining >2-fold above the background; an annexin V intensity >2-fold above the background across the “whole cell” region; nuclear collapse to small, homogenous, and intensely stained nuclear region (Hoechst channel); collapse of the nuclear/cytoplasm ratio; increasingly irregular cytoplasm shape; and increased formation of blebbing and/or apoptotic bodies. Possible fragmentation of the nucleus may occur, and care should be taken when segmenting nuclei in the Hoechst channel to ensure that oversegmentation does not occur.

##### Population Necrosis

Features of necrosis were intense Yo-Pro-3 staining within the “eroded nucleus” region, co-localized with Hoechst staining; absence of annexin V staining across the “whole cell” region; nuclear collapse to small, homogenous, and intensely stained nuclear region (Hoechst channel); collapse of the nuclear/cytoplasm ratio; increasingly irregular nuclear and cytoplasm shape with increased formation of apoptotic bodies; and dissociation of apoptotic bodies. Fragmentation of the nucleus may occur, and care should be taken when segmenting nuclei in the Hoechst channel to ensure that oversegmentation does not occur.

#### Classification 3: Supervised Classification

In our third classification technique, we performed supervised classification of single-cell measurements. Using the Cell Pathfinder software (Yokogawa), experienced cell biologists annotated cells from a reference experiment by eye as healthy, early apoptotic, late apoptotic, or necrotic/dead (**Suppl. Fig. S3A**). In order to enable easy categorization, we used a training set of compounds known to induce a specific, predominant modus of cell death. Staurosporine, gambogic acid, cisplatin, and paclitaxel served as inducers of apoptosis, whereas mercury chloride was used to induce necrosis (**Suppl. Fig. S3C**). However, after 24 h differentiating the phenotype between initial apoptotic and initial necrotic cells was challenging. Examples of healthy cells were chosen from both DMSO controls and cells treated with the CDK inhibitor GW276655 (**Suppl. Fig. S3B**). The latter was included as a reference for cell cycle arrest.

Each cell was divided into two objects, cell body and nucleus, with the requirement that each cell body contain at least one nucleus. The cell body was detected via digital phase contrast in addition to the fluorescent dyes. We extracted 16 features for the nucleus and 6 features for the cell body, including intensities of the dyes and morphology characteristics like size and roundness (**Suppl. Table S3**).

To validate our machine learning gating algorithm, we plotted the count of healthy cells against the logarithmic inhibitor concentrations and calculated IC_50_ values. Correct cell gating resulted in a decrease of the number of healthy cells and an increase of apoptotic and necrotic/dead-cell fractions in a dose-dependent manner. Interestingly, we noted different morphological features of apoptotic cells after treatment with different inhibitors. However, the gating algorithm was able to correctly assign these varying phenotypes as apoptotic or necrotic. In addition, by comparing the total cellular and nuclear area and the Hoechst intensity of healthy cells, we were able to observe a significantly increased cell body and nuclear area. This machine learning-guided approach proved to be easily adopted onto different adherent cell lines, highly accurate, and particularly user-friendly. These results confirm the validity of our approach in classifying cell death stages in different cell lines.

### Screening of Libraries and Hit Confirmation

**Figure 3.**
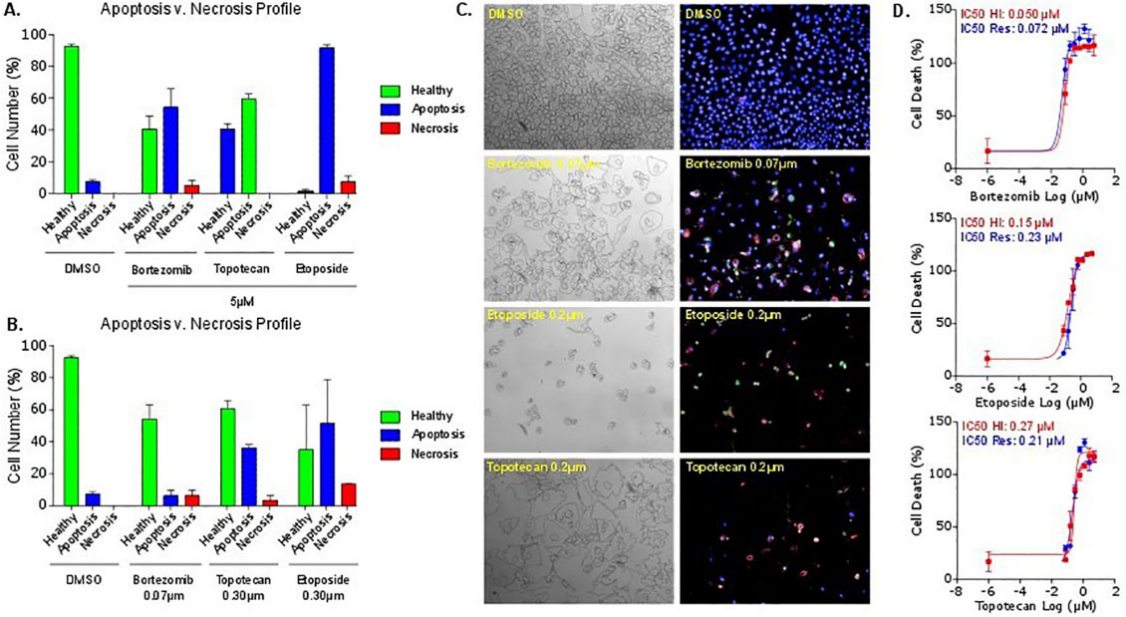
In vitro cytotoxic activity of three clinically approved anticancer drugs in AGP-01 after 72 h of exposure using population profiling—linear classifier. Percent of cells after treatment with control (DMSO) or bortezomib, topotecan, and etoposide, respectively, at (**A**) 5 µM or (**B**) IC_50_ concentration, in AGP-01 after 72 h of exposure. Early and late apoptosis populations have been combined for calculation. (**C**) Brightfield and high-content images with associated IC_50_ values, obtained by HCI and associated resazurin viability assay. (**D**) Quantification of small-compound cytotoxicity. Statistical significance was calculated in GraphPad Prism using an unpaired *t* test (*n* = 3). Any *p* values of ≤0.05 were considered statistically significant.

We next tested HighVia on different compounds. First, we used compounds from the FDA-approved oncology drug library, which exhibited an antiproliferative effect across four different cell lines in the resazurin assay using the Operetta and IN Cell 6000. These compounds were selected based on a range of antioncogenic mechanisms. A more detailed classification of cytotoxic effects is reflected in **Figure 3**, showing the in vitro cytotoxic activity of three anticancer compounds, the proteasome inhibitor bortezomib, the topoisomerase inhibitors topotecan and etoposide, and the DMSO control. Cytotoxicities at IC_50_ and 5 µM concentration are reported for AGP-01 (gastric cancer cell line) after 72 h of exposure. Although etopside and topotecan have similar IC_50_ values, the percentages of necrotic cells are very different between the two inhibitors, indicating different mechanisms of cell death.

As expected, there is overall a good correlation between the IC_50_ values determined in the resazurin assay compared with HighVia (**Fig. 3**). However, in cases of a less pronounced cytotoxic effect, or when the compound induces effects on cells different from apoptosis or necrosis, HighVia can provide additional information on the mechanism of cell death. An example is the analysis of methotrexate, an antimetabolite inhibiting dihydrofolate reductase. HighVia and resazurin result in IC_50_ values of 0.3 and 0.13 μM, respectively. Further interrogation of the high-content images shows a decreasing number of cells, and increasing nuclear and cell size as the concentration of methotrexate increases. In the case of methotrexate, this profiling may not be an entirely accurate reflection of the biological state. These cells were classified as “healthy,” as in this context we must consider “healthy” as not dead or dying. However, further analysis of profiled features of nuclear and cell size, as well as cell shape, revealed that these cells were not similar to the healthy control population, allowing them therefore to be more accurately classified as “senescent” in postclassification analysis.

Our protocol can accommodate a more detailed analysis of this feature. For example, senescence using SA-BGal,^15^ cell number, size and shape profiling, vesicle formation, and cycle arrest using nuclear Hoechst intensity could be integrated. **Supplemental Figure S6** shows clear vacuole formation using the suite of the three stains above with the addition of TMRM, obtained from Life Technologies. As mentioned above, TMRM is readily sequestered into active mitochondria, areas of vesicle formation appear as large voids in cytoplasm, and mitochondria are displaced.

**Figure 4.**
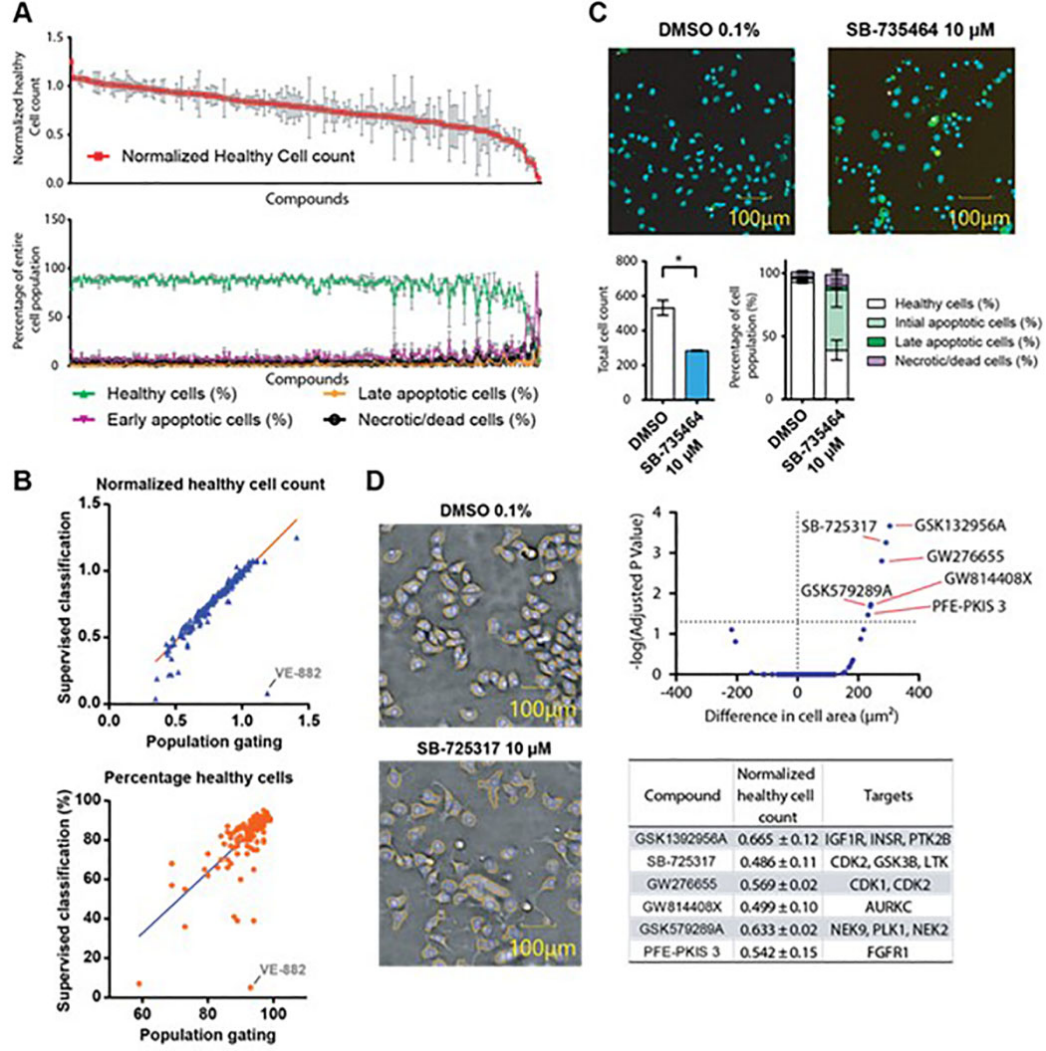
(**A**) Plotted healthy cell count normalized to the DMSO control and corresponding cellular fractions of PANC1 cells treated with the KCGS library at 10 µM for 24 h using the supervised classification method. (**B**) Comparison of the results of different gating mechanisms on selected inhibitors. Correlation of normalized healthy cell count (vs DMSO control) to the gated percentage of healthy cells of all tested 187 compounds of the both population gating and supervised classifications. Highlighted in both blots is the outlier VE-822. (**C**) Example of toxic compound, SB-735464, inducing apoptosis in PANC1 cells after 24 h with significantly reduced total cell count and increased apoptotic cell fractions. Student *t* test in prism with standard *p* values (*p < 0.05, **p < 0.01, *** *p* < 0.001, *n* = 3) using the supervised classification method. (**D**) Phase-contrast pictures of PANC1 cells incubated with DMSO or 10 µM SB-725317 for 24 h show enlarged cell bodies of the compound-treated cells (the detected cell body is depicted by the orange stroke). Volcano blot of difference in cell area of PANC1 cells of all compounds, with those that show a significant increase highlighted. These compounds were further analyzed with normalized cell count and annotated targets using the supervised classification method.

We further evaluated the developed method with a larger set of compounds using the CQ1. We utilized the KCGS available from the SGC. This is a set of 187 narrow-spectrum kinase inhibitors^16,17^ that can be used in phenotypic screens to help identify vulnerabilities of particular kinases to this set of inhibitors. We tested KCGS on the pancreatic cancer cell line PANC1. Cells were treated with the compound library at 10 µM for 24 h (**Fig. 4A**). As we aimed to detect different cell death modalities, a fairly high concentration of these potent kinase inhibitors was used. An early time point (24 h) was chosen in order to capture apoptotic events, rather than cell debris and correspondingly decreased cell numbers. To assess assay quality, we calculated the coefficients of variation between technical replicates of one biological replicate. Both normalized healthy count (to the average of healthy cells of DMSO wells) and the percentage of gated healthy cells were almost exclusively under 30% (**Suppl. Fig. S4**). We also used this test set of 187 compounds to compare the results of different gating methods. Gating results of the described population gating as well as of the supervised classification correlated generally well (**Fig. 4B**). Interestingly, the compound VE-822 led to a distinct phenotype of fragmented, intensely stained nuclei without annexin V or Yo-Pro-3 fluorescence. This feature was thus classified in the population gating as healthy, but the supervised classification gated this phenotype mainly as early apoptotic (**Suppl. Fig. S5**).

Most compounds in KCGS were relatively nontoxic, with only a few compounds leading to 20% or more unhealthy cells. A number of key features can be extracted from this experiment. For example, the compound SB-735464 showed increased numbers of cells in initial apoptosis compared with the DMSO control (**Fig. 4C**).

Including the cell area in the supervised analysis, compounds like GSK1392956A, an inhibitor for the kinases IGF1 R, INSR, and PTK2B, or SB-725317 and GW276655, both inhibiting CDKs, were identified (**Fig. 4D**). Inhibition of cell cycle regulators has been shown to result in enlarged cells in model organisms,^18^ but additional experiments are necessary to further evaluate the detailed mechanisms here.

In summary, we describe a high-throughput, high-content, multiplexed assay with a detailed analysis protocol. The protocols described are adaptable for use with multiple high-content readers and image analysis protocols—making the assay accessible to all laboratories with high-content imagers or to biomedical research scientists screening for cell viability/proliferation/cell death mechanisms through widely accessible academic screening facilities.^13,19^ Importantly, it is relatively inexpensive. Standard MTT assay costs are ∼£0.12/well, and those of HighVia are only 2.3× higher at ∼£0.28/well.

The toolbox of three fluorophores and transmitted light allows the detailed profiling of cellular viability and is applicable to multiple screening platforms including small compounds, as described here, in addition to siRNA and CRISPR platforms. Additional analysis parameters may be built into the analysis sequence, allowing further profiling and indications of additional cellular pathway inductions. The protocol is flexible and can be easily adapted to multiple cell types, whenever the cell segmentation protocols are robust. In addition to the cells described here, we have also validated the protocol in stem cells and neurons (unpublished data). Using HighVia, we can achieve a detailed understanding of the mechanism of cell death induced by libraries of test compounds, facilitating the search for novel and repurposed therapeutics. In addition to an IC_50_ value for compound toxicity, the image analysis offers additional insight into mechanisms of cell death, ranging from apoptosis to necrosis, as well as providing insight into additional compound effects, such as nuclear size and cell shape, thus allowing conclusions regarding the biological effect of the used compounds. Finally, these imaged data sets, once archived, can be reanalyzed to extract additional information if necessary.

## Supplemental Material

Supplemental Material, HighVia_Manuscript_Supplemental_FINAL_PUB - HighVia—A Flexible Live-Cell High-Content Screening Pipeline to Assess Cellular Toxicity Click here for additional data file. Supplemental Material, HighVia_Manuscript_Supplemental_FINAL_PUB for HighVia—A Flexible Live-Cell High-Content Screening Pipeline to Assess Cellular Toxicity by Alison Howarth, Martin Schröder, Raquel C. Montenegro, David H. Drewry, Heba Sailem, Val Millar, Susanne Müller and Daniel V. Ebner in SLAS Discovery

## References

[1] Zheng W. Thorne N. McKew J. C Phenotypic Screens as a Renewed Approach for Drug Discovery. Drug Discov. Today 2013, 18, 1067–1073. 23850704 10.1016/j.drudis.2013.07.001 PMC4531371

[2] Boutros M. Heigwer F. Laufer C Microscopy-Based High-Content Screening. Cell 2015, 163, 1314–1325. 26638068 10.1016/j.cell.2015.11.007

[3] Singh S. Carpenter A. E. Genovesio A. Increasing the Content of High-Content Screening: An Overview. J. Biomol. Screen. 2014, 19, 640–650. 24710339 10.1177/1087057114528537 PMC4230961

[4] Stoddart M. J. Cell Viability Assays: Introduction In Mammalian Cell Viability: Methods and Protocols, Stoddart M. J., Ed.; Humana Press: Totowa, NJ, 2011; pp 1–6. 10.1007/978-1-61779-108-6_1 21468961

[5] van Tonder A. Joubert A. M. Cromarty A. D Limitations of the 3-(4,5-Dimethylthiazol-2-yl)-2,5-Diphenyl-2*H*-Tetrazolium Bromide (MTT) Assay When Compared to Three Commonly Used Cell Enumeration Assays. BMC Res. Notes 2015, 8, 47. 25884200 10.1186/s13104-015-1000-8 PMC4349615

[6] Munshi S. Twining R. C. Dahl R Alamar Blue Reagent Interacts with Cell-Culture Media Giving Different Fluorescence over Time: Potential for False Positives. J. Pharmacol. Toxicol. Methods 2014, 70, 195–198. 24933394 10.1016/j.vascn.2014.06.005

[7] Vistica D. T. Skehan P. Scudiero D., et al. Tetrazolium-Based Assays for Cellular Viability: A Critical Examination of Selected Parameters Affecting Formazan Production. Cancer Res. 1991, 51, 2515. 2021931

[8] Hanahan D. Weinberg R. A Hallmarks of Cancer: The Next Generation. Cell 2011, 144, 646–674. 21376230 10.1016/j.cell.2011.02.013

[9] Bray M.-A. Singh S. Han H., et al. Cell Painting, a High-Content Image-Based Assay for Morphological Profiling Using Multiplexed Fluorescent Dyes. Nat. Protoc. 2016, 11, 1757–1774. 27560178 10.1038/nprot.2016.105 PMC5223290

[10] Chiaravalli J. Glickman J. F A High-Content Live-Cell Viability Assay and Its Validation on a Diverse 12 K Compound Screen. SLAS Discov. 2017, 22, 1120–1130. 28783477 10.1177/2472555217724745

[11] Timm M. Saaby L. Moesby L., et al. Considerations Regarding Use of Solvents in In Vitro Cell Based Assays. Cytotechnology 2013, 65, 887–894. 23328992 10.1007/s10616-012-9530-6 PMC3967611

[12] Kriel J. Loos B The Good, the Bad and the Autophagosome: Exploring Unanswered Questions of Autophagy-Dependent Cell Death. Cell Death Differ. 2019, 26, 640–652. 30659234 10.1038/s41418-018-0267-4 PMC6460391

[13] Shanks E. Ketteler R. Ebner D Academic Drug Discovery within the United Kingdom: A Reassessment. Nat. Rev. Drug Discov. 2015, 14, 510. 26091271 10.1038/nrd4661

[14] Thermo Fisher. Fluorescence SpectraViewer. https://www.thermofisher.com/uk/en/home/life-science/cell-analysis/labeling-chemistry/fluorescence-spectraviewer.html (accessed Dec 13, 2019).

[15] Chan K. T. Paavolainen L. Hannan K. M., et al. Combining High-Content Imaging and Phenotypic Classification Analysis of Senescence-Associated Beta-Galactosidase Staining to Identify Regulators of Oncogene-Induced Senescence. Assay Drug Dev. Technol. 2016, 14, 416–428. 27552145 10.1089/adt.2016.739

[16] Drewry D. H. Wells C. I. Zuercher W. J., et al. A Perspective on Extreme Open Science: Companies Sharing Compounds without Restriction. SLAS Discov. 2019, 24, 505–514. 31034310 10.1177/2472555219838210 PMC6624833

[17] Drewry D. H. Wells C. I. Andrews D. M., et al. Progress Towards a Public Chemogenomic Set for Protein Kinases and a Call for Contributions. *PLoS One* 2017, 12, e0181585. 28767711 10.1371/journal.pone.0181585 PMC5540273

[18] Amodeo A. A. Skotheim J. M Cell-Size Control. Cold Spring Harb. Perspect. Biol. 2016, 8, a019083–a. 26254313 10.1101/cshperspect.a019083 PMC4744813

[19] Frye S. Crosby M. Edwards T., et al. US Academic Drug Discovery. Nat. Rev. Drug Discov. 2011, 10, 409–410. 21629285 10.1038/nrd3462 PMC4461005

