# Supplementary material for: HighVia—A Flexible Live-Cell High-Content Screening Pipeline to Assess Cellular Toxicity
Source: SLAS Discov. 2020 May 27;25(7):801–11. doi: 10.1177/2472555220923979 (PMC7522770; doi:10.1177/2472555220923979)
Supplement: Supplemental Material, HighVia_Manuscript_Supplemental_FINAL_PUB - HighVia—A Flexible Live-Cell High-Content Screening Pipeline to Assess Cellular Toxicity [file HighVia_Manuscript_Supplemental_FINAL_PUB.pdf]

HighVia - A Flexible Live-cell High Content Screening Pipeline to Assess Cellular Toxicity

Howarth, A.[1†]; Schröder, M.[5†]; Montenegro, R.C. [2, 3†]; Drewry, D.H.[6]; Heba, S.[4]; Millar, V.[1]; Müller, S.[5]; Ebner, D.V.[1]

[1] Nuffield Department of Clinical Medicine, Target Discovery Institute, University of Oxford, Oxford, UK.

[2] Nuffield Department of Clinical Medicine, Structural Genomics Consortium, University of Oxford, Oxford, UK.

[3] Federal University of Ceará, Drug Research and Development Center (NPDM), Pharmacogenetics Laboratory, Fortaleza, CE, Brazil

[4] Department of Engineering, University or Oxford, Oxford, UK

[5] Structural Genomics Consortium, Buchmann Institute for Molecular Life Science, 60438 Frankfurt, Germany and Institute for Pharmaceutical Chemistry, Johann Wolfgang Goethe-University

[6] Structural Genomics Consortium, UNC Eshelman School of Pharmacy, University of North Carolina at Chapel Hill, Chapel Hill, North Carolina, USA

Supplemental Data

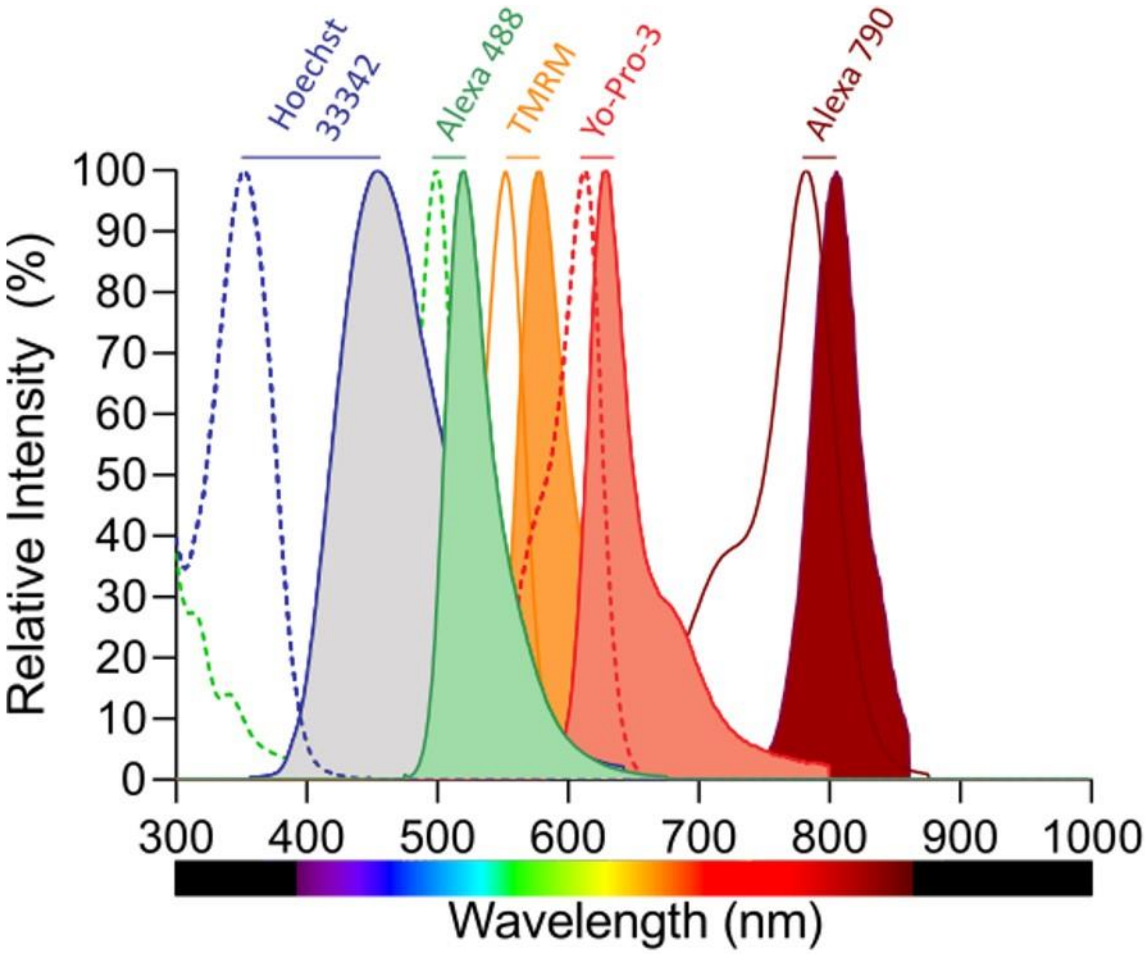

Supplemental Figure 1: Spectra Viewer visualisation of fluorophore excitation and emission wavelengths (https://www.thermofisher.com/uk/en/home/life-science/cell-analysis/labeling-chemistry/fluorescence-spectraviewer.html). Hoechst (blue), Annexin V- Alexa 488 (green) and Yo-Pro-3 (Red), as used in protocol. Additional fluorophores may be added in the regions highlighted, including Tetremethylrhodamine (TMRM) or far red fluorophores including Alexa-647 and -790.

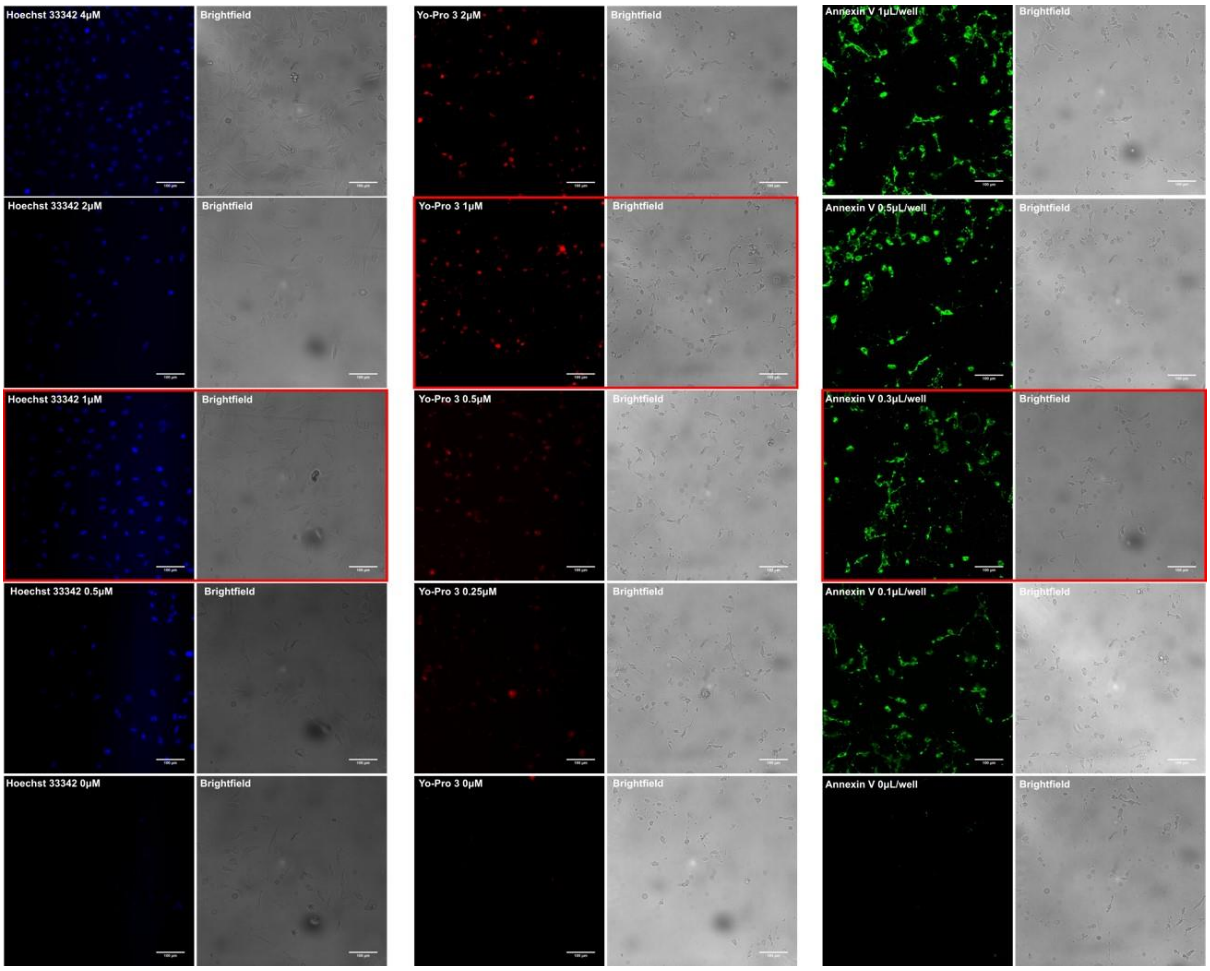

Supplemental Figure 2: Optimization of Hoechst (blue), Annexin V- Alexa 488 (green) and Yo-Pro-3 (Red) as used in the development of the HighVia protocol. Manufacturer's recommended concentration at the top of figure followed by two-fold serial dilutions. Hoechst 33342 - 1µM; Yo-Pro-3 – 1µM; and Annexin-V Alexa 488 - 0.3µL per well diluted in complete media yielded excellent signal to noise ratios and robust image segmentation for all high content imagers.

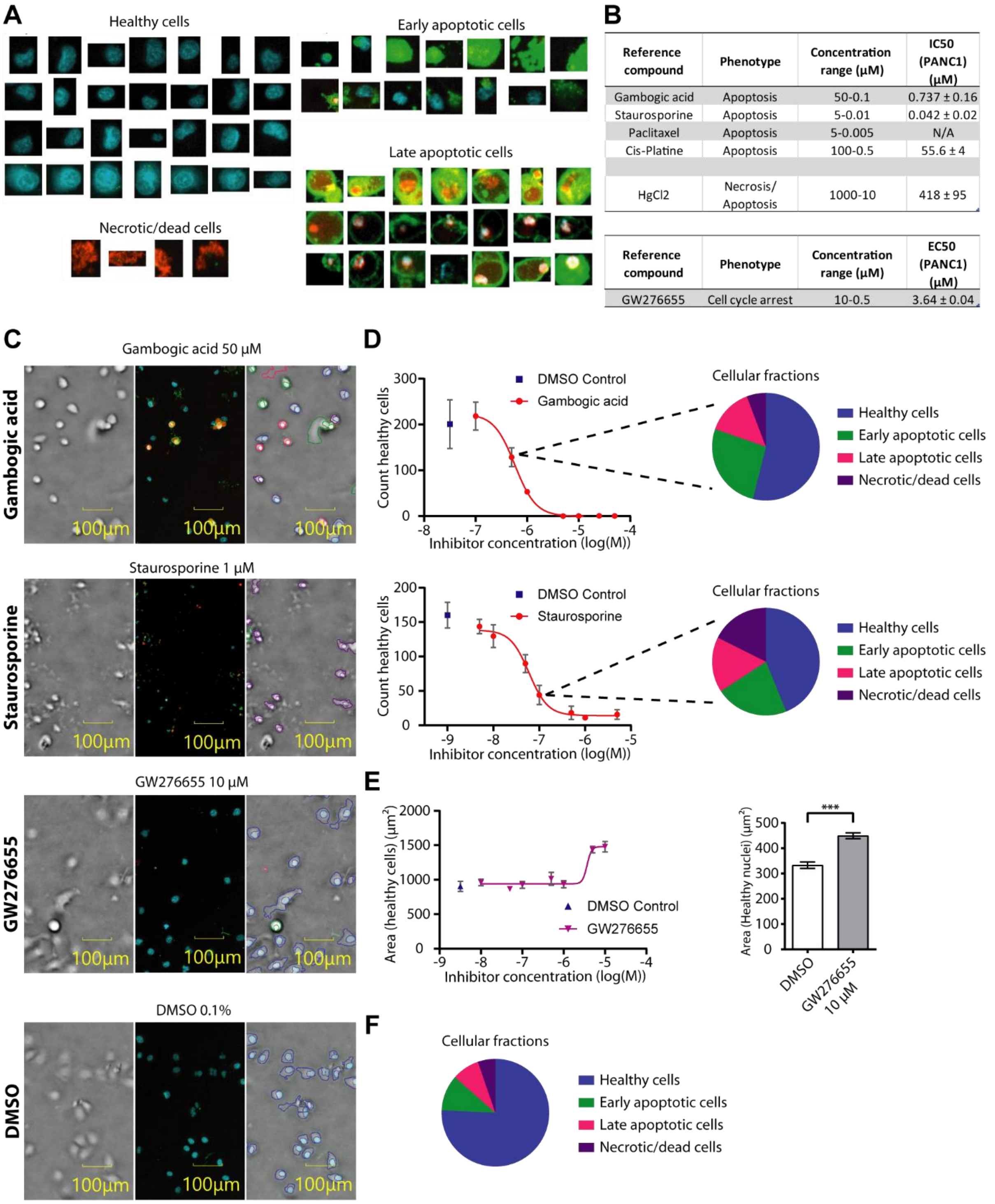

Reference compound	Phenotype	Concentration range (μM)	IC50 (PANC1) (μM)
Gambogic acid	Apoptosis	50-0.1	0.737 ± 0.16
Staurosporine	Apoptosis	5-0.01	0.042 ± 0.02
Paclitaxel	Apoptosis	5-0.005	N/A
Cis-Platine	Apoptosis	100-0.5	55.6 ± 4
HgCl2	Necrosis/ Apoptosis	1000-10	418 ± 95

Reference compound	Phenotype	Concentration range (μM)	EC50 (PANC1) (μM)
GW276655	Cell cycle arrest	10-0.5	3.64 ± 0.04

Supplemental Figure 3: Supervised gating mechanism using CQ1 (Yokogawa). **A**) Examples of cells used as training set for the machine learning algorithm. **B**) Table highlighting reference compounds with their corresponding phenotypes, recommended concentrations ranges and IC_{50}/EC_{50} values measured in PANC1 cells averaging data of two biological replicates. **C**) Examples of correctly gated PANC1

cells treated with different inhibitors or DMSO for 24h. Shown are bright field images with digital phase contrast, fluorescent pictures and merged pictures with gated cells. Detected cell bodies and nuclei are highlighted with a stroked outline and individual population are shown in different colors (blue: healthy cells, green: early apoptotic cells, late apoptotic cells: magenta, necrotic/dead cells: violet). **D**) Dose dependency of healthy PANC1 cell count and cellular area are shown for the corresponding inhibitors after 24h of treatment; analysis of cellular fractions of concentrations around the measured IC_{50} values. **E**) Plotted cellular area of PANC1 cells treated with different concentrations of GW276655 after 24h. Significant increase of nuclear area of PANC1 cells after 24h compared to DMSO control. Student's T test in prism with standard P values (* P<0.05, ** P<0.01, *** P<0.001, n=3) **F**) Cellular fractions of DMSO control after 24h.

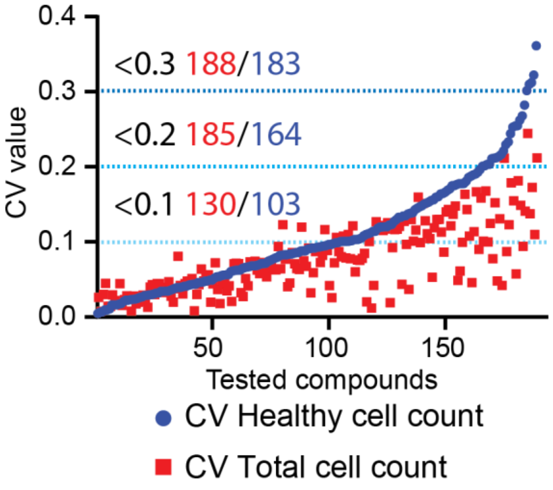

Supplemental Figure 4: CV values of replicates (n=3) of measured compounds using the supervised classification. Values were calculated with the SEM between technical replicates of one biological replicate.

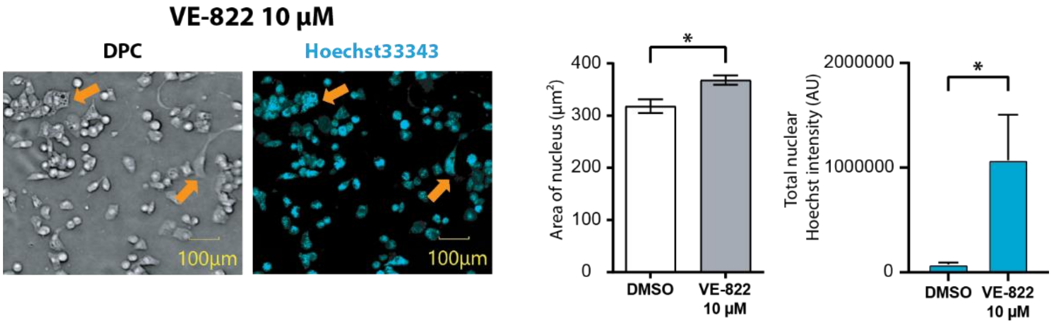

Supplemental Figure 5: PANC1 cells treated with 10 µM VE-822 show a phenotype of enlarged nuclei and intense Hoechst fluorescence. Orange arrows indicate the formation of vacuolar structures and diffuse, strong Hoechst signal within the PANC1 cells.

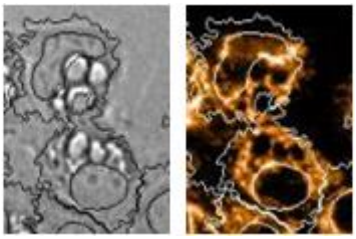

Supplemental Figure 6. In vitro cytotoxic effect of Estramustine, a clinically approved anticancer drug, in MCF-7 (breast cancer cell line) after 72hrs exposure. Cells show dramatic vacuole induction, visible in TMRM and brightfield channels. These cells display normal cytoplasm and nucleus morphology, but are EGFP and Yo-Pro-3 negative.

IC50 (µM)			
	**MDA-MB-231**	**HeLa**	**AGP-01**
**Vinorelbine**	0.0016	nd	0.28
**Mitoxantrone**	0.05	nd	0.088
**Cytarabine**	0.2	0.08	0.056
**Topotecan**	0.37	0.3	0.21
**Tenoposide**	3.05	0.10	0.39
**Methotrexate**	nd	0.0065	0.30
**Docetaxel**	0.21	0.63	0.52
**Bortezomib**	0.24	0.01	0.01
**Etoposide**	0.31	0.20	0.15
**Mitomycin**	0.01	0.0028	0.082

Supplemental Table 1: In vitro cytotoxic activity of 10 clinical approved anticancer drugs. IC_{50} values (µM) and confidence interval of 95% obtained by the resazurin assay in breast cancer cells (MDA-MB-231), cervical cancer cells (HeLa) and gastric cancer cells (AGP-01) after 72 hours of exposure. nd – not determined

	**DAPI/Hoechst 33342/CFP**	**FITC/GFP/Alexa-488**	**Yo-Pro-3 Alexa 633/647/CY5**
**Excitation (nm)**	360-400	460-490	620-640
**Emission (nm)**	410-480	500-550	650-670

Supplemental Table 2: Emission and excitation wavelengths of selected high content imaging fluorophores.

Cell Region	Feature (Parameter)
**Nuclear**	Size
**Nuclear**	Roundness
**Nuclear**	Total Intensity Hoechst (Ex:405nm Em:447/60; Z:MaxIP)
**Nuclear**	Total Intensity AnnexinV (Ex:488nm Em:525/50; Z:MaxIP)
**Nuclear**	Total Intensity YoPro (Ex:561nm Em:617/73; Z:MaxIP)
**Nuclear**	Mean Intensity Hoechst (Ex:405nm Em:447/60; Z:MaxIP)
**Nuclear**	Mean Intensity AnnexinV (Ex:488nm Em:525/50; Z:MaxIP)
**Nuclear**	Mean Intensity YoPro (Ex:561nm Em:617/73; Z:MaxIP)
**Nuclear**	Total Peak Energy Score Hoechst (Ex:405nm Em:447/60; Z:MaxIP; Mask Size: 5.0 µM)
**Nuclear**	Total Peak Energy Score AnnexinV (Ex:488nm Em:525/50; Z:MaxIP; Mask Size: 5.0 µM)
**Nuclear**	Total Ridge Energy Score Hoechst (Ex:405nm Em:447/60; Z:MaxIP; Mask Size: 5.0 µm)
**Nuclear**	Total Ridge Energy Score AnnexinV (Ex:488nm Em:525/50; Z:MaxIP; Mask Size: 5.0 µM)
**Nuclear**	Mean Peak Energy Score Hoechst (Ex:405nm Em:447/60; Z:MaxIP; Mask Size: 5.0 µM)
**Nuclear**	Mean Peak Energy Score AnnexinV (Ex:488nm Em:525/50; Z:MaxIP; Mask Size: 5.0 µM)
**Nuclear**	Mean Ridge Energy Score Hoechst (Ex:405nm Em:447/60; Z:MaxIP; Mask Size: 5.0 µM)
**Nuclear**	Mean Ridge Energy Score AnnexinV (Ex:488nm Em:525/50; Z:MaxIP; Mask Size: 5.0 µM)
**Cell body**	Size
**Cell body**	Roundness
**Cell body**	Total Intensity AnnexinV (Ex:488nm Em:525/50; Z:MaxIP)
**Cell body**	Total Intensity YoPro (Ex:561nm Em:617/73; Z:MaxIP)
**Cell body**	Mean Intensity AnnexinV (Ex:488nm Em:525/50; Z:MaxIP)
**Cell body**	Mean Intensity YoPro (Ex:561nm Em:617/73; Z:MaxIP)

Supplemental Table 3. Cell features used for supervised classification.
